# Supplementary material for: Hematological and biochemical profiles, infection and habitat quality in an urban rat population
Source: Sci Rep. 2025 Jul 22;15:26518. doi: 10.1038/s41598-025-09887-y (PMC12279998; doi:10.1038/s41598-025-09887-y)

Supplementary information

Supplementary Table S1: List of infection, environmental and biotic variables of the rats. These predictor variables were used to check for associations between them and rats’ health profiles.

| **Variable** | **Type** | **Levels** |  |  |
| --- | --- | --- | --- | --- |
| *Strongyloides* sp. infection | Binomial; Continuous | Presence/Absence; Intensity(log2(EPG)) |  |  |
| *N. brasiliensis* infection | Binomial; Continuous | Presence/Absence; Intensity(log2(EPG)) |  |  |
| *A. cantonensis* infection | Binomial; Continuous | Presence/Absence; Intensity(log2(EPG)) |  |  |
| *Hymenolepis* spp. infection | Binomial; Continuous | Presence/Absence; Intensity(log2(EPG)) |  |  |
| Trichiuridae infection | Binomial; Continuous | Presence/Absence; Intensity(log2(EPG)) |  |  |
| *L. interrogans* infection | Binomial; Continuous | Presence/Absence; Intensity(log2(EPG)) |  |  |
| Rat feces | Binomial | Presence/Absence |  |  |
| Trails | Binomial | Presence/Absence |  |  |
| Rat burrows | Continuous | Number of rat burrows |  |  |
| Geographic valley | Categorical | Valley 1 |  |  |
|  |  | Valley 2 |  |  |
|  |  | Valley 3 |  |  |
| Sex | Categorical | Female |  |  |
|  |  | Male |  |  |
| Age (d)^a^ | Continuous | Age in days |  |  |
| Maturity^b^ | Categorical | Immature |  |  |
|  |  | Mature |  |  |
| Scaled mass index (Smi)^c^ | Continuous | weight/length ratio index of overall body condition variable, which accounts for the effect of age |  |  |
| Internal fat | Binomial | Presence/Absence of visceral or subcutaneous fat |  |  |
| Wounds | Binomial | Presence/Absence of external wounds |  |  |
| ^a^ Panti-May et al., 2016 |  |  |  |  |
| ^b^ Carvalho-Pereira et al., 2017 | | |  |  |
| ^c^ Peig J, Green AJ. New perspectives for estimating body condition from mass/length data: the scaled mass index as an alternative method. Oikos 2009; 118:1883-1891. | | |  |  |

Supplementary Fig. S1 – Keiser-Guttman criterion for the assessment of the number of interpretable axes in PCA. A) Bc-PCA; B) Cort-PCA.


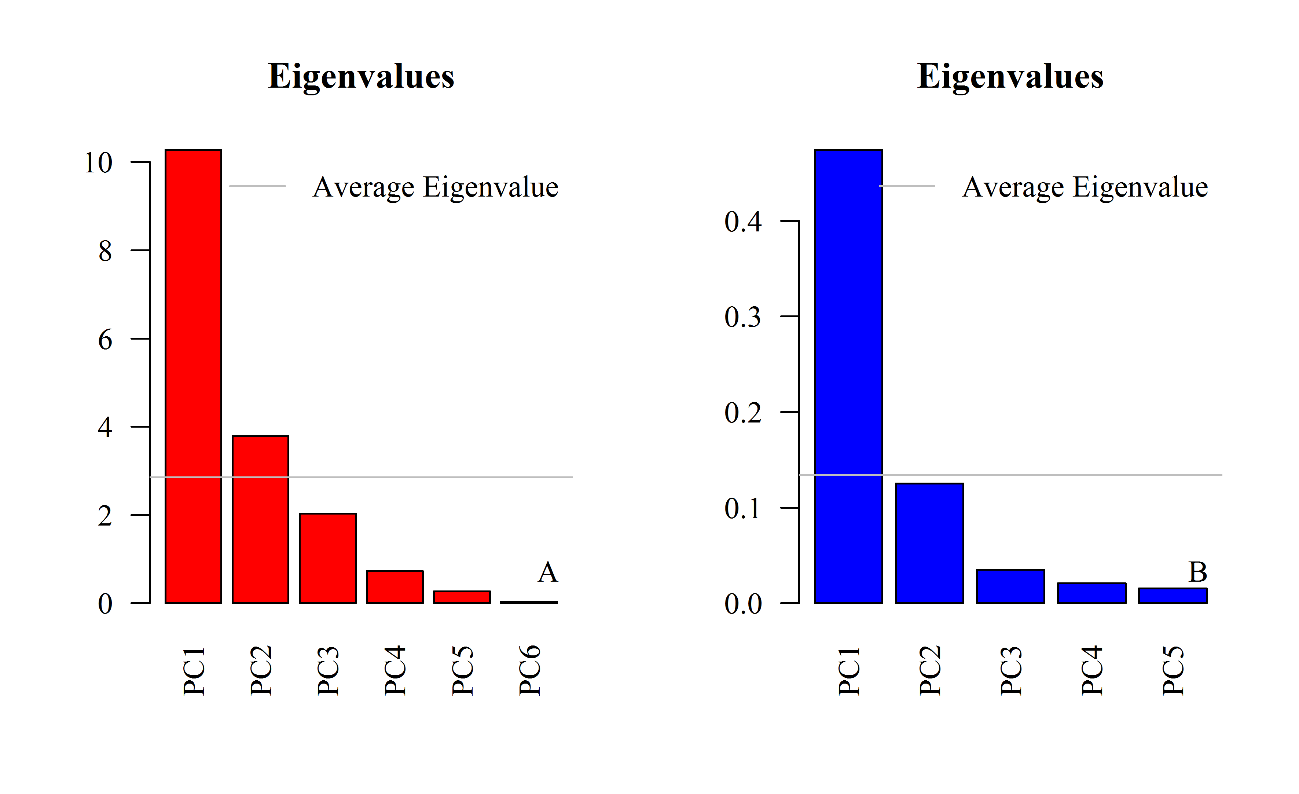


Supplementary Fig. S2 – PCA biplot (scaling 1) of the health profiles with overlaid clustering results. A) Bc-PCA clusters. B) Cort-PCA clusters.


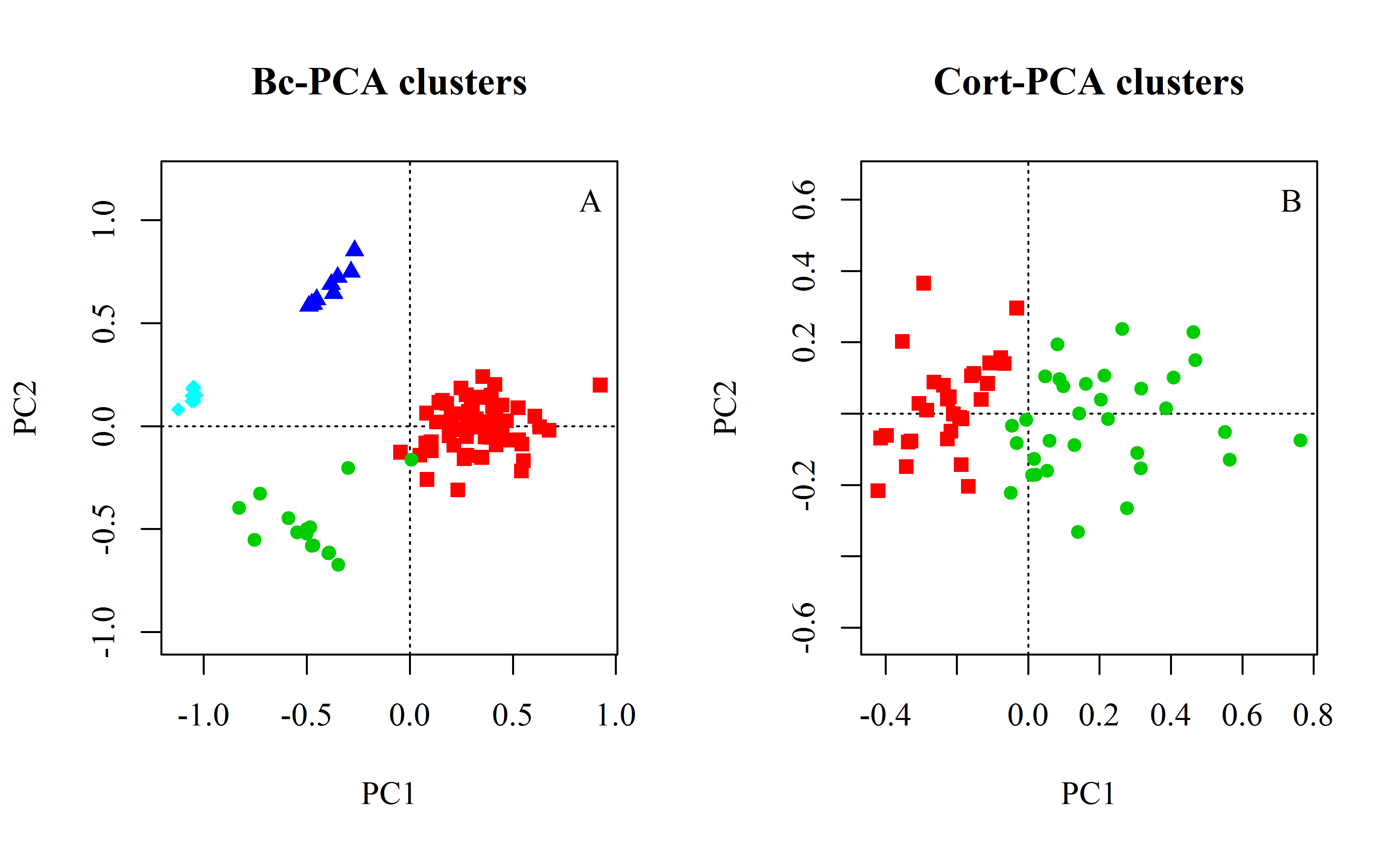

Supplement: Supplementary file 1 — Supplementary Material 1 [file 41598_2025_9887_MOESM1_ESM.docx]
